# Diagnostic performance and prognostic value of preoperative ^18^F-FDG PET/CT in renal cell carcinoma patients with venous tumor thrombus

**DOI:** 10.1186/s40644-022-00502-1

**Published:** 2022-11-26

**Authors:** Silu Chen, Yanyan Zhao, Qi Tang, Caixia Wu, Aixiang Wang, Linlin Ma, Xi Zhang, Jinzhi Chen, Yuan Gao, Xuhe Liao, Ninghan Feng, Yan Fan, Jianhua Zhang, Xuesong Li, Meng Liu

**Affiliations:** 1 Department of Nuclear Medicine, Peking University First Hospital, No.8, Xishiku Street, West District, Beijing, 100034 China; 2 Department of Urology, Peking University First Hospital, Institute of Urology, Peking University, National Urological Cancer Center, No.8, Xishiku Street, West District, Beijing, 100034 China; 3 Department of Urology, Wuxi No. 2 People’s Hospital, Wuxi, 214002 China

**Keywords:** ^18^F-FDG PET/CT, Renal cell carcinoma (RCC), Venous tumor thrombus (VTT), Maximum standardized uptake value (SUVmax), Prognosis

## Abstract

**Background:**

To observe the diagnostic efficacy of preoperative fluorine-18 fluorodeoxyglucose (^18^F-FDG) positron emission tomography/computed tomography (PET/CT) upon venous tumor thrombus (VTT) in patients with renal cell carcinoma (RCC), and investigate the prognostic value of imaging parameters integrated with clinicopathological characteristics in patients with VTT after nephrectomy with tumor thrombectomy.

**Methods:**

Patients with newly diagnosed RCC who underwent ^18^F-FDG PET/CT were reviewed retrospectively. The diagnostic efficacy of ^18^F-FDG PET/CT in VTT was analyzed. Logistic regression analysis was carried out to identify the clinical variables and PET/CT variables (including maximum standardized uptake value (SUVmax) of primary tumor, VTT SUVmax and primary tumor size) for differentiating early VTT (Mayo 0-II) from advanced VTT (Mayo III-IV). Cox proportional hazard analyses were used to evaluate clinicopathological factors and PET/CT factors (including distant metastasis, primary tumor SUVmax, VTT SUVmax and primary tumor size) for disease-free survival (DFS) in patients with VTT after operation.

**Results:**

A total of 174 eligible patients were included in this study, including 114 men (65.5%) and 60 women (34.5%), with a median age of 58 years (range, 16–81 years). The distribution of pathological tumor stage (T stage) was 56 (T1), 17 (T2), 95 (T3), and 6 cases (T4), respectively. According to WHO/ISUP grade, except for 4 cases of chromophobe cell RCC, there were 14 patients (8.0%) of grade 1, 59 patients (33.9%) of grade 2, 74 patients (42.5%) of grade 3 and 23 patients (13.2%) of grade 4. The median maximum diameter of the primary tumor on PET/CT was 7.3 cm (5.0–9.5 cm). The distal metastasis was observed in 46 patients (26.4%). Sixty-one cases (35.1%) were confirmed with VTT by pathology. The sensitivity, specificity, accuracy, positive predictive value, and negative predictive value of ^18^F-FDG PET/CT imaging were 96.7, 99.1, 98.3, 98.3, and 98.2%, in detecting VTT, respectively, and 70.0, 100.0, 94.9, 100.0, and 94.2%, in evaluating the level of VTT, respectively. Elevated VTT SUVmax (≥5.20) could significantly distinguish the early VTT group and advanced VTT group (*P* = 0.010). In the prognosis analysis, elevated VTT SUVmax (≥4.30) (*P* = 0.018, HR 3.123, 95% CI 1.212–8.044) and distant metastasis (*P* = 0.013, HR 3.344, 95% CI 1.293–8.649) were significantly independent predictors for DFS.

**Conclusion:**

Preoperative ^18^F-FDG PET/CT has a high diagnostic efficacy in detecting VTT and evaluating its level in RCC patients. Those patients with elevated VTT SUVmax should be carefully monitored to detect the possibility of disease progression after operation.

## Background

Approximately 4–10% of patients with renal cell carcinoma (RCC) have venous tumor thrombus (VTT), which is one of the significant adverse prognostic factors [1]. Nephrectomy with tumor thrombectomy is suggested as the most common management for RCC patients with VTT, with the 5-year survival rate reaching up to 57–72% [2, 3]. VTT above hepatic veins (Mayo III-IV) is likely to require cardiac surgeons to operate with cardiopulmonary bypass or venovenous bypass on the same stage [4]. Therefore, according to differences in surgical procedure, VTT is recommended to be divided into early VTT (Mayo 0-II) and advanced VTT (Mayo III-IV) [5–9]. Since the presence of VTT and its level may affect the formulation of treatment strategies [10], it is vitally important to accurately diagnose VTT and evaluate its level prior to surgery.

Currently, magnetic resonance imaging (MRI) or contrast-enhanced computed tomography (CECT) is widely used in the preoperative evaluation of venous thrombi [11, 12]. In comparison with MRI and CECT, fluorine-18 fluorodeoxyglucose (^18^F-FDG) positron emission tomography/computed tomography (PET/CT), as a systemic functional molecular imaging method, can not only virtually cover the region of VTT but also have obvious advantages in differentiating the benign and malignant thrombus from the glucose metabolic perspective [13].

VTT is mainly composed of malignant tumor cells and presents an increased uptake of ^18^F-FDG compared with benign thrombus (BT) that consists of platelets, fibrin mesh, and macrophages [14]. Sharma, P et al. proposed that the maximum standardized uptake value (SUVmax) of VTT was significantly higher than that of BT in a variety of malignant tumors, including hepatocellular carcinoma (HCC), non-Hodgkin’s lymphoma, and RCC [14]. Additionally, our previous study demonstrated that the presence of VTT and elevated SUVmax of primary lesion from preoperative ^18^F-FDG PET/CT can effectively distinguish high World Health Organization/International Society of Urological Pathology (WHO/ISUP) grade of clear cell renal cell carcinoma (ccRCC) [15]. But to the best of our knowledge, reports are lacking in the diagnostic performance and prognostic value of preoperative ^18^F-FDG PET/CT in RCC patients with VTT.

In this study, we tried to observe the diagnostic efficacy of preoperative ^18^F-FDG PET/CT upon VTT in patients with RCC, and investigate the prognostic value of imaging parameters integrated with clinicopathological characteristics in patients with VTT after nephrectomy with tumor thrombectomy.

## Material and methods

### Patient characteristics

For patients with renal tumors who were highly suspected of distant metastasis, ^18^F-FDG PET/CT would be performed for a systemic oncological assessment in our hospital. The electronic medical records of consecutive RCC patients who underwent ^18^F-FDG PET/CT examination prior to surgery from March 2014 to August 2021 were retrospectively reviewed.

**Fig. 1:**
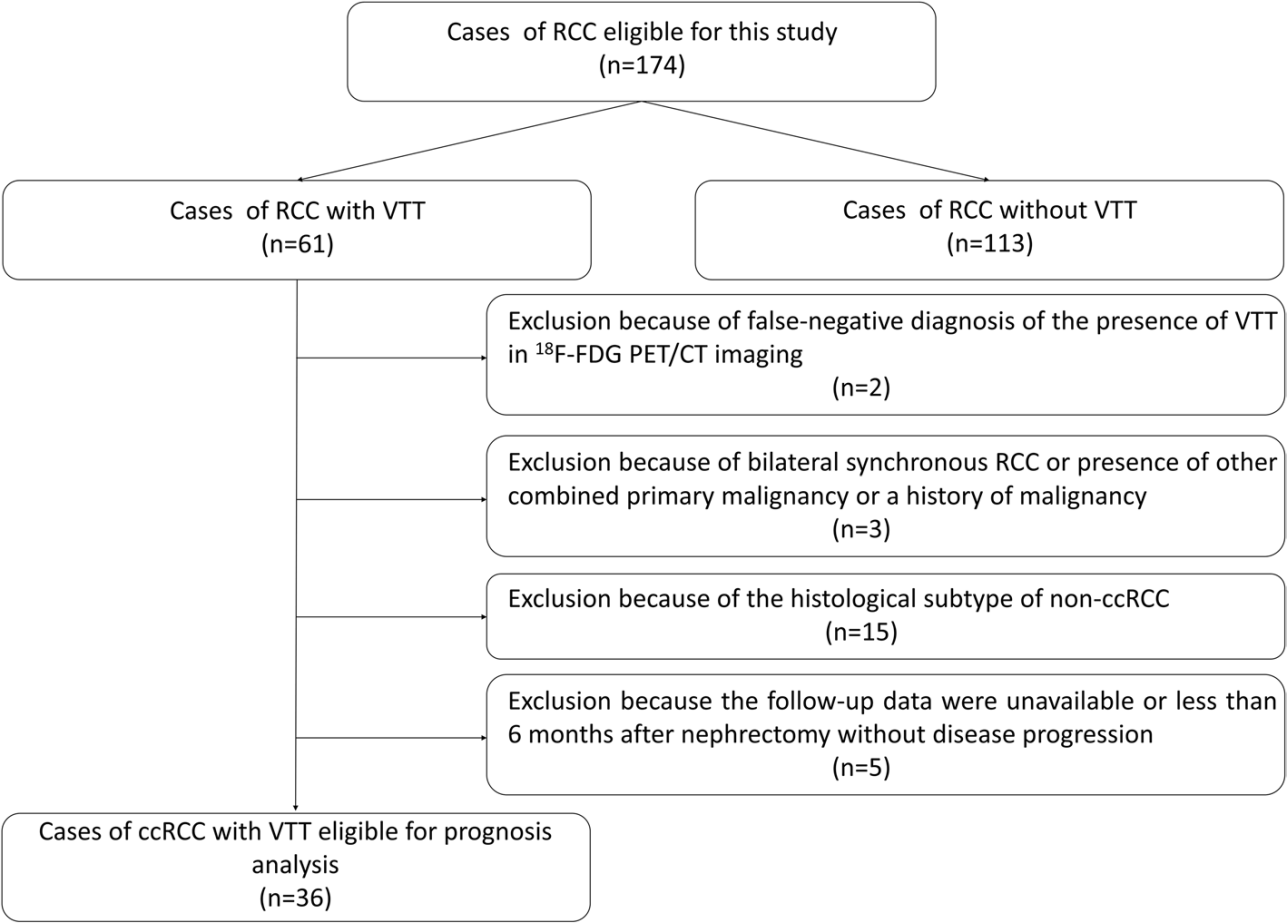
The flow diagram about the inclusion and exclusion of patients

The inclusion criteria were as follows [15–17]: (1) newly diagnosed RCC by primary tumor pathological analyses, (2) radical nephrectomy or combined with tumor thrombectomy (for patients with VTT) performed at our hospital, and (3) ^18^F-FDG PET/CT performed before operation and systematic treatment initiation. The additional inclusion criterion for prognosis analysis was available follow-up data for more than 6 months after operation for the patients without disease progression. The additional exclusion criteria for prognosis analysis were as follows: (1) patients with false-negative diagnosis of presence of VTT in ^18^F-FDG PET/CT imaging, (2) patients with histological subtype of non-ccRCC, (3) patients with presence of other combined primary malignancy or a history of malignancy including RCC, and (4) patients with bilateral synchronous RCC. The flow diagram of the inclusion and exclusion of patients is presented in Fig. 1.

The documented clinicopathological parameters included age, gender, blood glucose, body mass index (BMI), symptoms, histological subtype, and WHO/ISUP grade. Patients with hematuria, abdominal mass, abdominal distension, abdominal/waist/back pain, nausea, fatigue, fever, weight loss, and metastasis as first symptoms (including cough, sputum with blood, bone pain, and so on) were considered symptomatic [18, 19]. The presence or absence of VTT was determined by experienced genitourinary pathologists. Based on the Mayo clinic classification system, the levels of VTT were classified into early VTT group (Mayo 0-II) and advanced group (Mayo III-IV), and confirmed by experienced senior urologists according to intraoperative findings.

Patient records were anonymized and deidentified before analysis. The retrospective data collection and analysis procedures were approved by the Ethics Committee of our hospital, waiving the need for written informed consent.

### Imaging analysis of ^18^F-FDG PET/CT

As described in our previous studies [15], preoperative ^18^F-FDG PET/CT images were acquired. Two experienced senior nuclear medicine physicians, who were unaware of the patients’ information, evaluated the images independently. If they came to different viewpoints, the third blinded senior nuclear medicine physician would evaluate the image to reach a consensus.

Referring to our previous studies [15], according to the PET/CT images, with reference to contrast-enhanced CT or MRI if necessary, we measured the primary tumor SUVmax by carefully delineating a volume of interest (VOI). The VOI was carefully put on the primary lesion to encompass the tumor as much as possible with the minimum physiological activity of the renal calyces. VTT was diagnosed according to the abnormal accumulation of ^18^F-FDG in the renal vein or inferior vena cava, which was higher than that in the abdominal aorta at the same level. A region of interest (ROI) was outlined for measuring the SUVmax of VTT [20]. The tumor size was expressed as the maximum diameter line of the primary tumor on PET/CT. The regional lymph node and/or distant metastases were evaluated in line with the eighth edition of the American Joint Committee on Cancer (AJCC) TNM staging system [8].

### Follow up and clinical endpoint

As depicted previously [17], follow-up surveillance after surgery included abdomen ultrasonography or abdomen CT, chest X-ray, and laboratory data, which were regularly collected every 3 months for the first 2 years, then every 6 months until the fifth year, and annually afterward. Disease-free survival (DFS) was defined as the date from operation to recurrence and/or metastasis proven by radiology or pathology, death of any cause, or censored at the last follow-up [17]. Recurrence was defined as locoregional recurrence or progression of the initial distant metastases according to the Response Evaluation Criteria in Solid Tumor (RECIST) guideline (version 1.1) [16].

### Statistical analysis

The diagnostic efficacy of ^18^F-FDG PET/CT for VTT and its level was expressed as sensitivity, specificity, accuracy, positive predictive value (PPV), and negative predictive value (NPV). The kappa test was employed to estimate the consistency between imaging results and pathologically or clinically confirmed results for the presence or level of VTT, respectively.

Continuous variables were shown as the mean ± SD or medians (first quartile-third quartile, Q1-Q3), and categorical variables were shown as numbers (percentages). Student’s *t* test was used to compare the age, BMI and primary tumor size between the advanced VTT group and early VTT group. Mann–Whitney 𝑈 test was used to compare primary tumor SUVmax and VTT SUVmax between the advanced VTT group and early VTT group, and was used to compare VTT SUVmax between different groups divided by histological subtype, WHO/ISUP grade, level of VTT and distant metastasis. Fisher’s exact test was used to compare the gender and symptoms between advanced VTT group and early VTT group. Receiver operating characteristic (ROC) curves were generated for the optimal cutoff value and area under the curve (AUC) for the continuous variables of primary tumor size and SUVmax. Spearman rank correlation test was used to confirm the linear correlation between VTT SUVmax and primary tumor SUVmax.

Univariate logistic regression analysis was carried out to identify the variables associated with early VTT and advanced VTT. The continuous variables were dichotomized into disease-free and disease-progression groups, using the cutoff values by the receiver operating characteristic (ROC) curve analysis. Univariate and multivariate Cox proportional hazard analyses were used to evaluate potential prognostic factors for DFS, and the hazard ratios (HR) and 95% confidence intervals (CI) of the predictors were acquired. Survival analysis was assessed by Kaplan-Meier curves, and the log-rank test was employed to compare the survival rates.

Statistical analyses were executed using SPSS 26.0 software (SPSS Software Inc., Chicago, IL, USA) and GraphPad Prism 8.0 software (GraphPad Software Inc., La Jolla, CA, USA). *P* < 0.05 were deemed statistically significant.

## Results

### General characteristics

As shown in Table 1, one hundred and seventy-four patients with newly diagnosed RCC were included in this study, including 114 men (65.5%) and 60 women (34.5%), with a median age of 58 years (range 16–81 years). The distribution of pathological tumor stage (T stage) included 56 cases (32.2%) of T1, 17 cases (9.8%) of T2, 95 cases (54.6%) of T3, 6 cases (3.4%) of T4. For histological subtypes, 137 cases (78.7%) were ccRCC, and 37 cases (21.3%) were non-ccRCC. According to WHO/ISUP grade, except for 4 cases of chromophobe cell RCC, there were 14 patients (8.0%) of grade 1, 59 patients (33.9%) of grade 2, 74 patients (42.5%) of grade 3 and 23 patients (13.2%) of grade 4. The median maximum diameter of the primary tumor on PET/CT was 7.3 cm (5.0–9.5 cm). The distal metastasis was observed in 46 patients (26.4%).

**Table 1:** General characteristics of RCC patients

Characteristics	Value
No. of patients	174
Age (ys)	
median	58
range	16–81
Gender *n* (%)	
Male	114 (65.5)
Female	60 (34.5)
Blood glucose (mmol/L)	6.0 (5.5–6.7)
BMI (kg/m^2^)	24.88 ± 3.55
Symptoms *n (%)*	
Presence	105 (60.3)
Absence	69 (39.7)
Pathological T stage *n (%)*	
T1	56 (32.2)
T2	17 (9.8)
T3	95 (54.6)
T4	6 (3.4)
Histological subtype *n* (%)	
ccRCC	137 (78.7)
Papillary RCC	17 (9.8)
Clear-cell papillary RCC	6 (3.4)
Chromophobe RCC	4 (2.3)
Xp11.2 translocation/TFE3 gene fusion RCC	4 (2.3)
Succinate dehydrogenase-deficiency RCC	2 (1.1)
Others	4 (2.3)
WHO/ISUP grade *n* (%)	
G1	14 (8.0)
G2	59 (33.9)
G3	74 (42.5)
G4	23 (13.2)
Chromophobe cell RCC not available for WHO/ISUP grade	4 (2.3)
Primary tumor size (cm)	7.3 (5.0–9.5)
Distal metastasis *n* (%)	
Presence	46 (26.4)
Absence	128 (73.6)

Abbreviations: *RCC* renal cell carcinoma, *BMI* body mass index, *ccRCC* clear cell renal cell carcinoma, *WHO/ISUP* World Health Organization/the International Society of Urological Pathology

Thirty-six ccRCC patients were included for prognostic analysis, including 29 males (80.6%) and 7 females (19.4%), with a median age of 57 years (50–66 years). The median follow-up time was 13.6 months (range: 1.3–74.1 months), and the median progression time was 27.5 months. Sixteen patients experienced tumor progression, and two patients died, accounting for 50.0% of the total cases. Nine patients (25.0%) received adjuvant therapy after the operation.

### Diagnostic value of ^18^F-FDG PET/CT imaging for VTT and its level

Among the 174 enrolled patients, the number of cases with VTT confirmed by pathology was 61 (35.1%). The diagnostic performance of ^18^F-FDG PET/CT imaging on the presence of VTT are shown in Table 2. The sensitivity, specificity, accuracy, PPV, and NPV were 96.7, 99.1, 98.3, 98.3, and 98.2%, respectively. Kappa value between PET/CT and clinically confirmed results was 0.962 in diagnosing the presence of VTT. One false-positive case showed mild widening of renal vein and slight accumulation of ^18^F-FDG, and two false-negative cases due to the too tiny VTT to be found.

**Table 2:** Diagnostic value of ^18^F-FDG PET/CT in the presence of VTT

Histopathology	PET/CT imaging		Total
	Presence of VTT	Absence of VTT	
With VTT	59	2	61
Without VTT	1	112	113
Total	60	114	174

Abbreviations: *PET/CT* positron emission tomography/computed tomography, *VTT* venous tumor thrombus

**Fig. 2:**
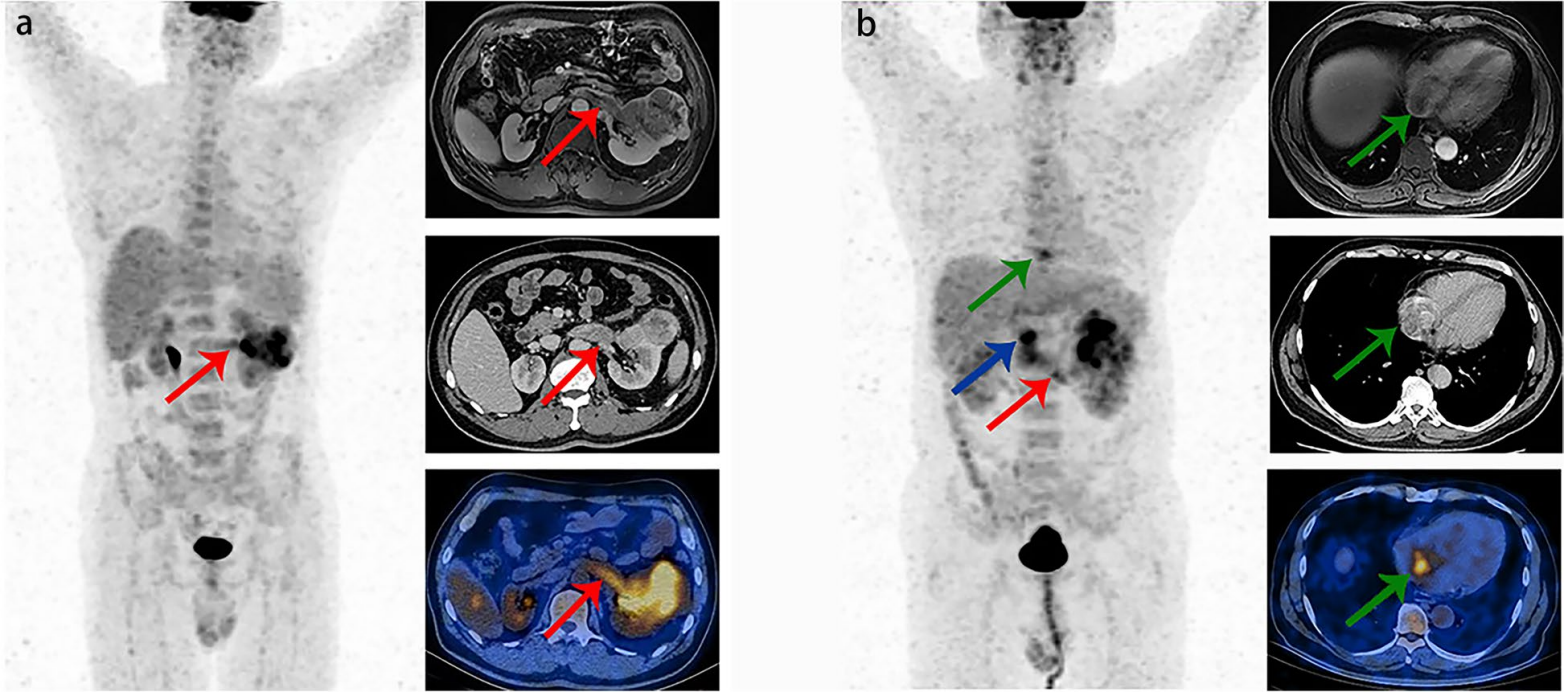
**a** Images in a 57-year-old male with ccRCC demonstrate left renal vein tumor thrombus (red arrows), with VTT SUVmax of 4.5 (left image: anterior maximum intensity projection image obtained at ^18^F-FDG PET; right upper image: axial contrast-enhanced MRI image; right middle image: axial contrast-enhanced CT image; right lower image: axial fused PET/CT image). **b** Images in a 55 year-old male with ccRCC demonstrate left renal vein tumor thrombus (red arrow), inferior vena cava tumor thrombus (blue arrow), and tumor thrombus in the right atrium (green arrows), with VTT SUVmax of 5.7 (left image: anterior maximum intensity projection image obtained at ^18^F-FDG PET; right upper image: axial contrast-enhanced MRI image; right middle image: axial contrast-enhanced CT image; right lower image: axial fused PET/CT image)

As shown in Table 3, the diagnostic value of ^18^F-FDG PET/CT imaging for the level of VTT (early VTT or advanced VTT) was further analyzed in 59 true-positive cases with VTT, including 49 early VTT and 10 advanced VTT. The sensitivity, specificity, accuracy, PPV, and NPV were 70.0, 100.0, 94.9, 100.0, and 94.2%, respectively. Kappa value between PET/CT and clinically confirmed results was 0.795 in diagnosing the level of VTT. The typical cases are shown in Fig. 2.

**Table 3:** Diagnostic value of ^18^F-FDG PET/CT in the level of VTT

Intraoperative finding	PET/CT imaging		Total
	Early VTT	Advanced VTT	
Early VTT	49	0	49
Advanced VTT	3	7	10
Total	52	7	59

Abbreviations: *PET/CT* positron emission tomography/computed tomography, *VTT* venous tumor thrombus

### Correlations of PET/CT parameters and clinical characteristics with the level of VTT

The characteristics of 59 true-positive cases with VTT are summarized in Table 4. In univariate logistic regression analysis, VTT SUVmax was the only factor that could dramatically distinguish early VTT from advanced VTT (*P* = 0.010, HR 1.336, 95% CI 1.073–1.664). In the ROC curve analysis, the cutoff value of VTT SUVmax to differentiate the early VTT group and advanced VTT group was 5.20, with a sensitivity of 90.0% and specificity of 75.5% (*P* = 0.002, AUC = 0.820).

**Table 4:** Correlations of ^18^F-FDG PET/CT parameters and clinical characteristics with the level of VTT

Characteristic	Total (***n*** = 59)	Advanced VTT (***n*** = 10)	Early VTT (***n*** = 49)	***P*** value
**Clinical parameters**				
Age (ys)	54.8 ± 12.8	56.0 ± 15.6	54.6 ± 12.3	0.750^a^
Gender				0.254^c^
Male	42(71.2%)	9(90.0%)	33(67.3%)	
Female	17(28.8%)	1(10.0%)	16(32.7%)	
Symptoms				0.333^c^
Presence	50(84.7%)	10(100.0%)	40(81.6%)	
Absence	9(15.3%)	0(0.0%)	9(18.4%)	
BMI (kg/m^2^)	25.2 ± 3.6	25.2 ± 2.9	25.2 ± 3.8	0.999^a^
**PET/CT parameters**				
Primary tumor SUVmax	6.6(4.3–10.4)	8.7(5.9–10.7)	6.0(4.2–10.8)	0.203^b^
VTT SUVmax	3.7(2.5–6.1)	6.5(5.3–8.8)	3.4(2.5–5.4)	**0.002**^**b**^
Primary tumor size (cm)	8.8 ± 2.5	9.4 ± 3.3	8.7 ± 2.4	0.450^a^

Continuous variables (age, BMI, primary tumor SUVmax, VTT SUVmax and primary tumor size) are expressed as the median (first quartile-third quartile, Q1-Q3) or mean value ± SD. Categoric variables (gender, symptoms) were expressed as numbers (percentages). *P* value < 0.05 was highlighted using bold font

Abbreviations: *VTT* venous tumor thrombus, *BMI* body mass index, *SUVmax* maximum standardized uptake value

^a^Student’s t test

^b^Mann-Whitney U test

^c^Fisher’s exact test

### Correlations of VTT SUVmax with clinicopathological characteristics and other PET/CT parameters

**Fig. 3:**
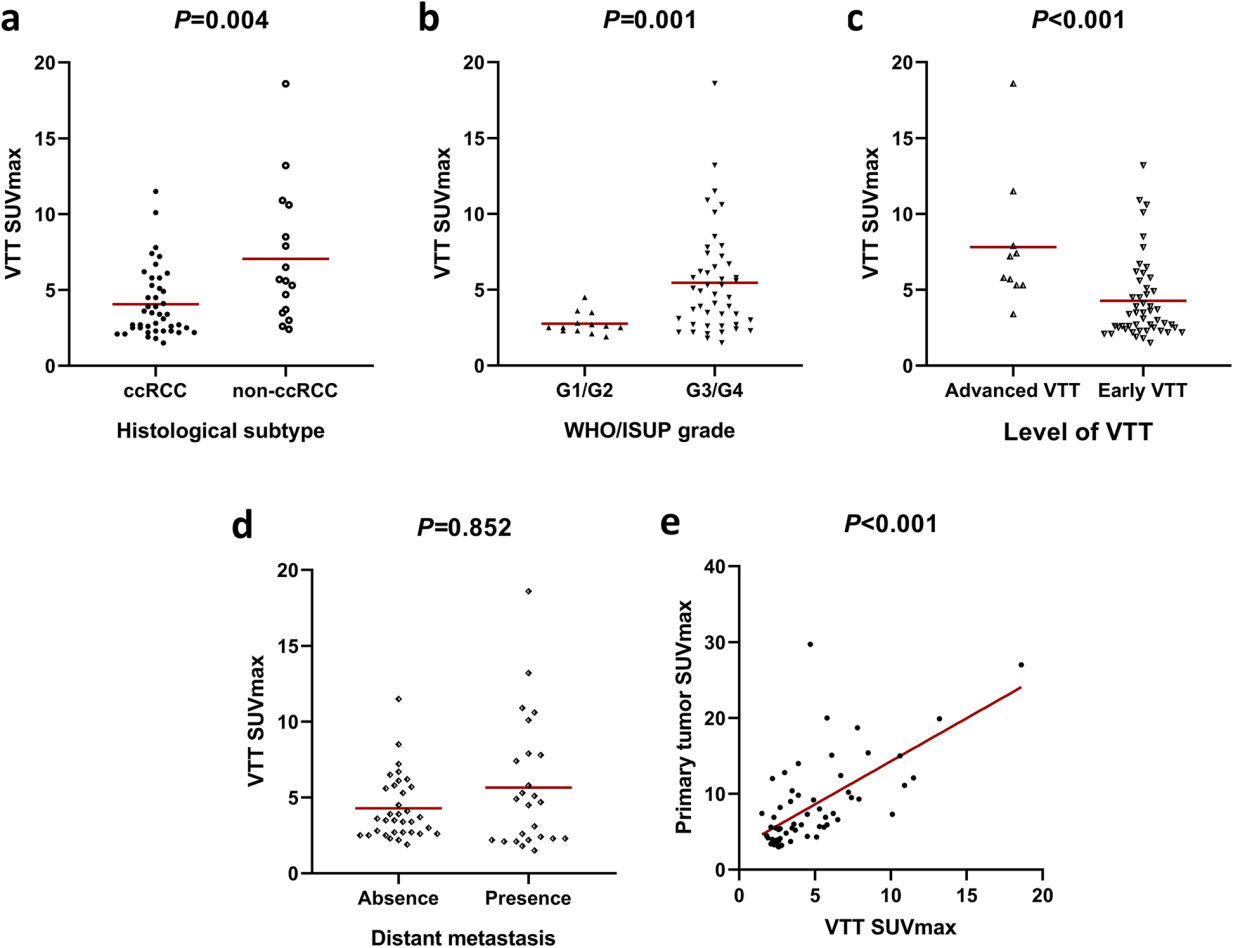
Comparisons of VTT SUVmax between subtypes of histological subtype (**a**), WHO/ISUP grade (**b**), level of VTT (**c**) and distant metastasis (**d**), and correlation with primary tumor SUVmax (**e**), respectively

As presented in Fig. 3, VTT SUVmax was markedly higher in patients with non-ccRCC (*P* = 0.004), WHO/ISUP grade 3/4 (*P* = 0.001), and advanced VTT (*P <* 0.001), but had no remarkable difference between the metastasis and non-metastasis groups (*P* = 0.852). A significant linear correlation was found between VTT SUVmax and primary tumor SUVmax (*P* < 0.001, r = 0.667).

### Prognostic factor analysis

Considering the differences in prognosis of various histological subtypes [21–23], only ccRCC patients with VTT were enrolled in prognostic analysis. As determined by ROC curves, the cutoff value of primary tumor SUVmax to predict DFS was 4.45, with a sensitivity of 77.8% and specificity of 55.6%, and that of VTT SUVmax was 4.30, with a sensitivity of 44.4% and specificity of 83.3%. The cutoff value for primary tumor size was 8.65 cm determined by median. The results of univariate and multivariate Cox proportional hazards analysis are shown in Table 5.

**Table 5:** Cox proportional hazards analysis for disease-free survival of ccRCC patients with VTT after nephrectomy and thrombectomy

Variable	Number(%)	Univariate			Forward Stepwise Multivariate		
		HR	95% CI	*P* value	HR	95% CI	*P* value
**Clinicopathological parameters**							
Age (years)				0.329			
≥ 60	16(44.4)	0.619	0.237–1.620				
< 60	20(55.6)	1.000(ref.)					
Gender				0.352			
Male	29(80.6)	0.580	0.184–1.828				
Female	7(19.4)	1.000(ref.)					
Symptoms				0.510			
Presence	31(86.1)	1.642	0.376–7.180				
Absence	5(13.9)	1.000(ref.)					
BMI (kg/m^2^)				0.896			
≥ 24.00 kg/m^2^	28(77.8)	0.928	0.304–2.834				
< 24.00 kg/m^2^	8(22.2)	1.000(ref.)					
Level of VTT				0.802			
Advanced	5(13.9)	1.174	0.337–4.090				
Early	31(86.1)	1.000(ref.)					
WHO/ISUP grade				*0.038*			
G3/G4	25(69.4)	4.760	1.092–20.742				
G1/G2	11(30.6)	1.000(ref.)					
**PET/CT parameters**							
Distant metastasis				*0.012*			*0.013*
Presence	14(38.9)	3.382	1.307–8.751		3.344	1.293–8.649	
Absence	22(61.1)	1.000(ref.)			1.000(ref.)		
Primary tumor SUVmax				0.081			
≥ 4.45	22(61.1)	2.704	0.885–8.255				
< 4.45	14(38.9)	1.000(ref.)					
VTT SUVmax				*0.017*			*0.018*
≥ 4.30	11(30.6)	3.167	1.227–8.174		3.123	1.212–8.044	
< 4.30	25(69.4)	1.000(ref.)			1.000(ref.)		
Primary tumor size (cm)				0.634			
≥ 8.65	18(50.0)	0.797	0.313–2.030				
< 8.65	18(50.0)	1.000(ref.)					

Abbreviations: *ccRCC* clear cell renal cell carcinoma, *BMI* body mass index, *VTT* venous tumor thrombus, *WHO/ISUP* World Health Organization/the International Society of Urological Pathology, *PET/CT* positron emission tomography/computed tomography, *SUVmax* maximum standardized uptake value

*P* value < 0.05 was highlighted using italic font

**Fig. 4:**
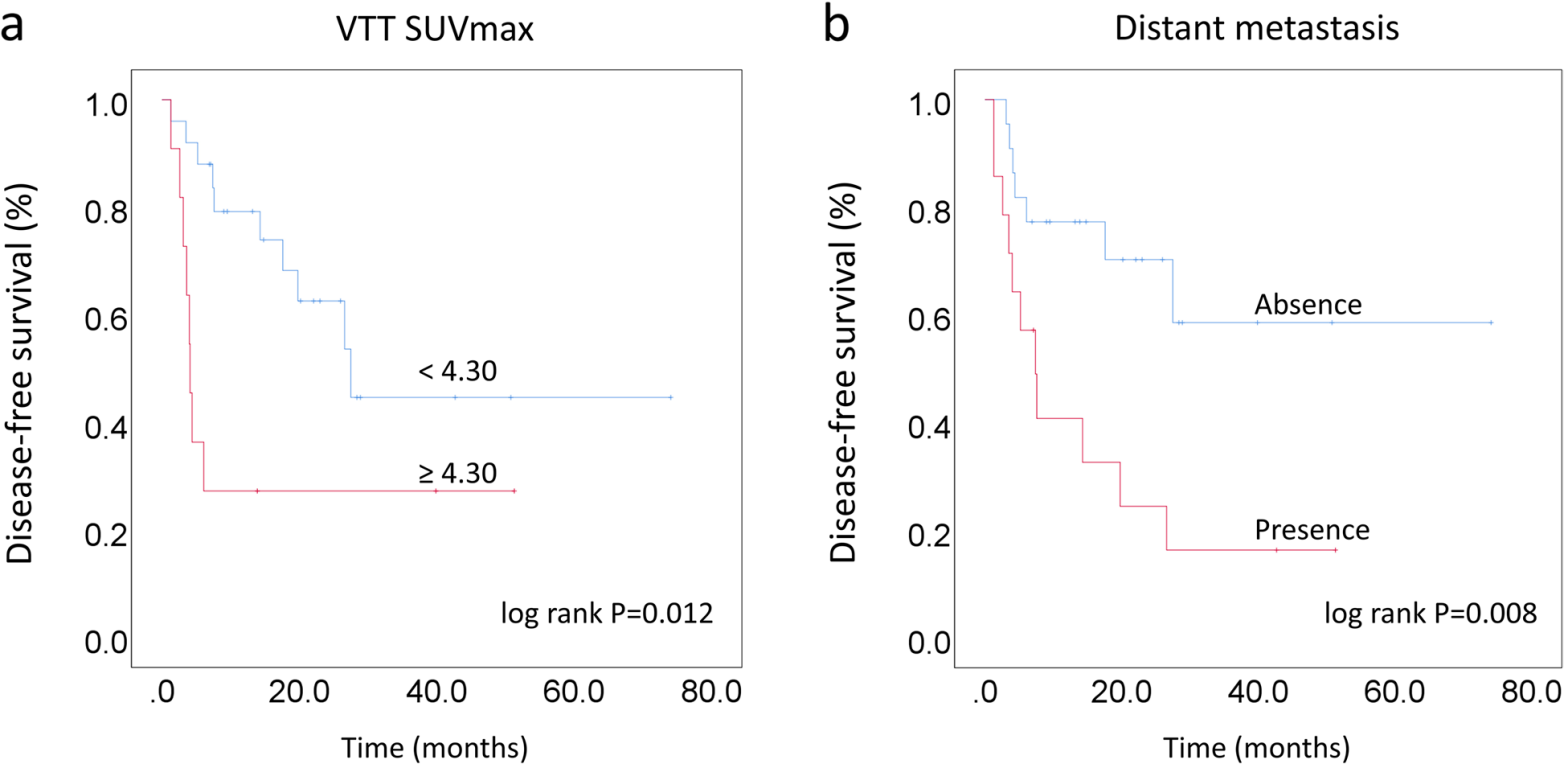
Kaplan-Meier survival curves analyses for DFS between the groups categorized according to VTT SUVmax (**a**) and distant metastasis (**b**)

**Fig. 5:**
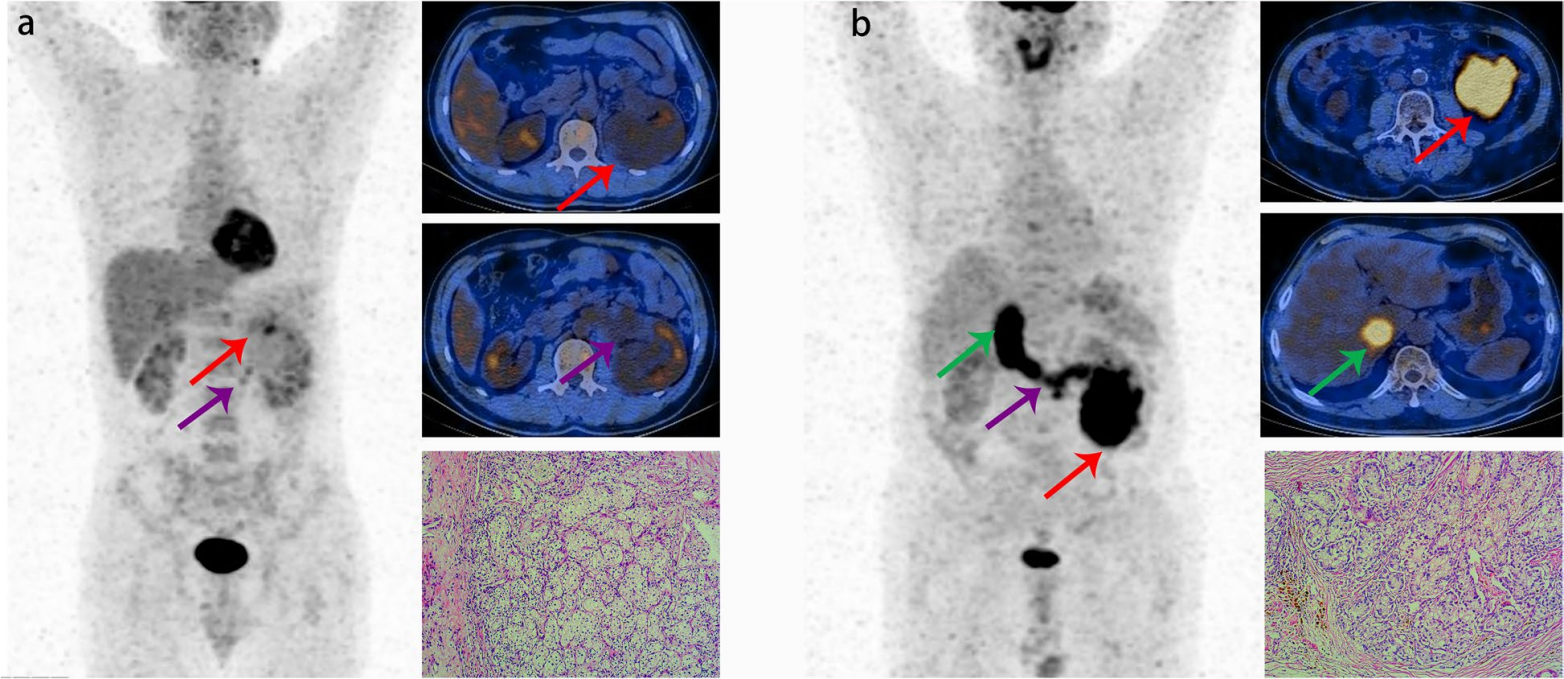
**a** Images in a 44-year-old male with ccRCC demonstrate the primary lesion in the left kidney (red arrows) with SUVmax of 3.0, WHO/ISUP grade 2, and left renal vein tumor thrombus (purple arrows) with SUVmax of 2.6 (left image: anterior maximum intensity projection image obtained at ^18^F-FDG PET; right upper image: axial fused PET/CT image of primary lesion; right middle image: axial fused PET/CT image of renal vein tumor thrombus; right lower image: H&E result of primary lesion). No distant metastasis was identified. The patient remained in the state of disease-free survival after the radical nephrectomy with tumor thrombectomy with a follow-up of 29 months. **b** Images in a 64-year-old male with ccRCC demonstrate the primary lesion in the left kidney (red arrows) with SUVmax of 12.1, WHO/ISUP grade 3, and vein tumor thrombus above hepatic veins (purple and green arrows) with SUVmax of 11.5 (left image: anterior maximum intensity projection image obtained at ^18^F-FDG PET; right upper image: axial fused PET/CT image of primary lesion; right middle image: axial fused PET/CT image of vein tumor thrombus; right lower image: H&E result of primary lesion). Left lumbar vein tumor thrombus was also observed. The patient progressed after the radical nephrectomy and removal of inferior vena cava thrombus with a follow-up of 3 months

Elevated VTT SUVmax (*P* = 0.018, HR 3.123, 95% CI 1.212–8.044) and distant metastasis (*P* = 0.013, HR 3.344, 95% CI 1.293–8.649) from ^18^F-FDG PET/CT were found to be independent predictors for DFS. Kaplan-Meier survival curves showed that in ccRCC patients with VTT, those accompanied with elevated VTT SUVmax (≥4.30) (*P* = 0.012) and distant metastasis (*P* = 0.008) had more unfavorable DFS than their counterparts (Fig. 4). The typical cases are shown in Fig. 5**.**

## Discussion

Accurate diagnosis of VTT and preoperative evaluation of its level directly affect the formulation of treatment strategy and even the preparation of multidisciplinary surgical team cooperation [10]. VTT might be effectively evaluated by comprehensive information from ^18^F-FDG PET/CT, including abnormal morphology and increased glucose metabolism in the vein. However, the diagnostic performance and prognostic value of ^18^F-FDG PET/CT in RCC patients with VTT are not well known.

In our study, ^18^F-FDG PET/CT presented well in sensitivity, specificity, and accuracy. One false-positive case showed mild widening of renal vein and slight accumulation of ^18^F-FDG, which might be caused by inflammatory changes of renal veins [24]. As for the two false-negative cases, the pathological analysis showed the VTTs were too tiny, so the VTT were quite hard to be found in ^18^F-FDG PET/CT images. Furthermore, three cases of advanced VTT were mistaken as early VTT in ^18^F-FDG PET/CT images because FDG uptake of VTT section above hepatic vein was inapparent. In addition, the previous research demonstrated that the ^18^F-FDG PET/CT showed satisfying performance in grading VTT, although it was less effective than contrast enhanced MRI [25].

The preoperative characterization of VTT includes evaluating its level, which will affect the choice of surgical method. We found that VTT SUVmax (≥5.20), but not primary tumor SUVmax, was a significant predictive factor for differentiating advanced VTT from early VTT. We speculated that the rapid extension of VTT requires more glucose as energy, resulting in high uptake of ^18^F-FDG. Although VTT is most commonly seen in solid tumors adjacent to large veins, such as RCC or HCC, its pathophysiology remains poorly understood. Wang X et al. proposed that the tumor thrombosis process was a predetermined event that might be associated with genetic mutations of BAP1 or SETD2 in primary tumors [26]. Interestingly, we demonstrated that the FDG uptake of VTT was higher in cases with non-ccRCC subtypes, WHO/ISUP grade 3/4, and advanced VTT. Moreover, there was a significant linear correlation between VTT SUVmax and primary tumor SUVmax. The conglomerates of these findings suggested that the higher glucose metabolic activity of VTT may reflect the more aggressive characteristics of RCC.

With respect to the prognosis of ccRCC patients with VTT after surgery, we indicated that elevated VTT SUVmax and distant metastasis from ^18^F-FDG PET/CT were the reliable parameters for DFS in both the univariate and multivariate Cox analyses. One characteristic of ccRCC is its propensity to invade the renal vein or inferior vena cava, which results in the formation of VTT. Previous studies mainly focused on the prognostic value of the existence of VTT in RCC patients, and the conclusions were inconsistent [16, 27, 28]. Our study clarified that VTT SUVmax rather than primary tumor SUVmax was an independent prognostic factor for ccRCC patients with VTT after operation, which may be explained that the glucose metabolic behavior of VTT has a great impact on the prognosis in these patients. As another prognostic factor shown in this study, distant metastasis has also been confirmed as an independent prognostic factor in untreated RCC patients with VTT [29] and postoperative RCC patients with VTT [5, 30]. Whether the level of VTT could be a prognostic predictor in RCC patients with VTT remains controversial [5, 31–33]. Our results elucidated that there was no significant difference in postoperative prognosis between advanced VTT group and early VTT group.

As for WHO/ISUP grade, it was significant in univariate cox proportional hazards analysis, but was not sufficient to predict DFS independently in the multivariate Cox analysis here. The possible reason might be that when the WHO/ISUP grade was incorporated in the current research, its predictive weight was weakened by VTT SUVmax and distant metastasis with more value on prognosis. Besides, the tumor size is critical for patients with T stage of T2 or below. However, the presence of VTT was a landmark for T3 or above, and the tumor size was not a determinant of T stage for T3 or above patients. Therefore, compared to distant metastasis and VTT SUVmax, tumor size may not be a critical prognostic factor for such relatively advanced stage patients.

Admittedly, there are several important considerations in our research. First of all, this is a retrospective and single-center study, especially the small sample size in the prognostic analysis cohort, so inherent biases are inevitable. A prospective study should be designed in a larger population or in multicenter cohorts to validate the reliability of our findings. Second, in addition to evaluating the level of VTT, whether VTT invades or adheres to the venous wall is also a critical issue. The application of artificial intelligence and imaging omics may be a good choice in the future work. Last but not least, based on the meaningful results in the current study, it is necessary to further explore the potential mechanism of glucose metabolic reprogramming in primary lesion and VTT, which will help to better understand the characteristics and prognosis of RCC.

## Conclusions

This research illustrated that preoperative ^18^F-FDG PET/CT imaging had a high diagnostic efficacy in detecting VTT and evaluating its level in patients with RCC. Meanwhile, elevated VTT SUVmax may distinguish the level of VTT, which could provide useful information for the formulation of operative strategy. Those patients with elevated VTT SUVmax should be carefully monitored to detect the possibility of disease progression after nephrectomy with tumor thrombectomy as early as possible in the routine clinical practice.

## Data Availability

The datasets of current study are available from the corresponding author on reasonable request.

## Abbreviations

^18^F-FDG: Fluorine-18 fluorodeoxyglucose
AUC: Area under the receiver operating characteristic curve
ccRCC: Clear cell renal cell carcinoma
DFS: Disease-free survival
PET/CT: Positron emission tomography/computed tomography
RCC: Renal cell carcinoma
SUVmax: Maximum standardized uptake value
VTT: Venous tumor thrombus
WHO/ISUP: World Health Organization/the International Society of Urological Pathology
